# A molecular epidemiological and transmission analysis of *Clostridioides difficile* using draft whole-genome sequencing in a single hospital

**DOI:** 10.1186/s12879-024-09841-9

**Published:** 2024-09-17

**Authors:** Taito Miyazaki, Kotaro Aoki, Tadashi Maeda, Kohji Komori, Sadako Yoshizawa, Yoshikazu Ishii, Yoshihisa Urita, Kazuhiro Tateda

**Affiliations:** 1 Infection Control Section, Toho University Omori Medical Center, Tokyo, Japan; 2 Department of General Medicine and Emergency Care, Toho University School of Medicine, Tokyo, Japan; 3 Department of Microbiology and Infectious Diseases, Toho University School of Medicine, 5-21-16 Omori-nishi, Ota-ku, Tokyo, 143-8540 Japan; 4 Department of Microbiology and Infection Control and Prevention, Toho University Graduate School of Medicine, Tokyo, Japan; 5 Department of Laboratory Medicine, Faculty of Medicine, Toho University School of Medicine, Tokyo, Japan; 6 Center for the Planetary Health and Innovation Science (PHIS), The IDEC Institute, Hiroshima University, Higashi-Hiroshima, Japan

**Keywords:** *Clostridioides difficile*, Whole-genome sequencing, SNP analysis, Silent transmission

## Abstract

**Background:**

The nosocomial transmission of toxin-producing *Clostridioides difficile* is a significant concern in infection control. *C. difficile*, which resides in human intestines, poses a risk of transmission, especially when patients are in close contact with medical staff.

**Methods:**

To investigate the nosocomial transmission of *C. difficile* in a single center, we analyzed the genetic relationships of the bacteria. This was done using draft whole-genome sequencing (WGS) and examining single nucleotide polymorphisms (SNPs) in core-genome, alongside data regarding the patient’s hospital wards and room changes. Our retrospective analysis covered 38 strains, each isolated from a different patient, between April 2014 and January 2015.

**Results:**

We identified 38 strains that were divided into 11 sequence types (STs). ST81 was the most prevalent (*n* = 11), followed by ST183 (*n* = 10) and ST17 (*n* = 7). A cluster of strains that indicated suspected nosocomial transmission (SNT) was identified through SNP analysis. The draft WGS identified five clusters, with 16 of 38 strains belonging to these clusters. There were two clusters for ST81 (ST81-SNT-1 and ST81-SNT-2), two for ST183 (ST183-SNT-1 and ST183-SNT-2), and one for ST17 (ST17-SNT-1). ST183-SNT-1 was the largest SNT cluster, encompassing five patients who were associated with Wards A, B, and K. The most frequent room changer was a patient labeled Pt08, who changed rooms seven times in Ward B. Patients Pt36 and Pt10, who were also in Ward B, had multiple admissions and discharges during the study period.

**Conclusions:**

Additional culture tests and SNP analysis of *C. difficile* using draft WGS revealed silent transmission within the wards, particularly in cases involving frequent room changes and repeated admissions and discharges. Monitoring *C. difficile* transmission using WGS-based analysis could serve as a valuable marker in infection control management.

**Supplementary Information:**

The online version contains supplementary material available at 10.1186/s12879-024-09841-9.

## Background

*Clostridioides difficile* is a Gram-positive, spore-forming, anaerobic bacterium and a pathogen responsible for *C. difficile* infection (CDI) [1]. The occurrence of CDI, caused by toxin A or B-producing *C. difficile*, is concerning not only due to adverse patient outcomes but also due to the increased costs associated with treatment and infection control [2]. In particular, the spore-forming nature of *C. difficile* necessitates different decontamination methods compared to those used for general bacteria and requires more labor-intensive care for CDI patients [2–4]. Tracking the transmission of *C. difficile* between patients and interrupting transmission pathways is crucial for infection prevention in hospitals [5].

*C. difficile* typing is typically performed using PCR ribotyping (RT) [6, 7]. Strains that belong to RT027 are known for causing CDI with severe symptoms or high mortality rates [8, 9]. With the widespread availability of whole-genome sequencing (WGS) facilitated by massive parallel sequencers, the typing of *C. difficile* has shifted from RT to methods such as multilocus sequencing typing (MLST), core-genome MLST, and single nucleotide polymorphism (SNP)-based typing [10–13]. These sequence-based typing methods have proven to be robust for analysis in fields beyond nosocomial transmission, including food poisoning and One-Health [14, 15]. Notably, SNPs-based typing is considered the best practice for distinguishing bacterial strains.

The enzyme immunoassays (EIA) and nucleic acid amplification testing (NAAT) for detecting *C. difficile* and its Toxin A/B antigens in stool samples are commonly used for diagnosing CDI [1, 16]. Routine *C. difficile* culture tests are often omitted because EIA and NAAT exhibited good concordance with culture test results, take less time, and the culture method requires an additional toxin or toxin-encoding gene detection test, which takes 48 h or more [17]. Consequently, *C. difficile* strain collections with clinical information are valuable for nosocomial transmission analysis using WGS [18].

In this retrospective study, we aimed to detect silent nosocomial transmissions of *C. difficile* during the study period by integrating WGS and SNPs-based analyses with epidemiological data from the hospital wards.

## Materials and methods

### Patients and strains

**Fig. 1:**
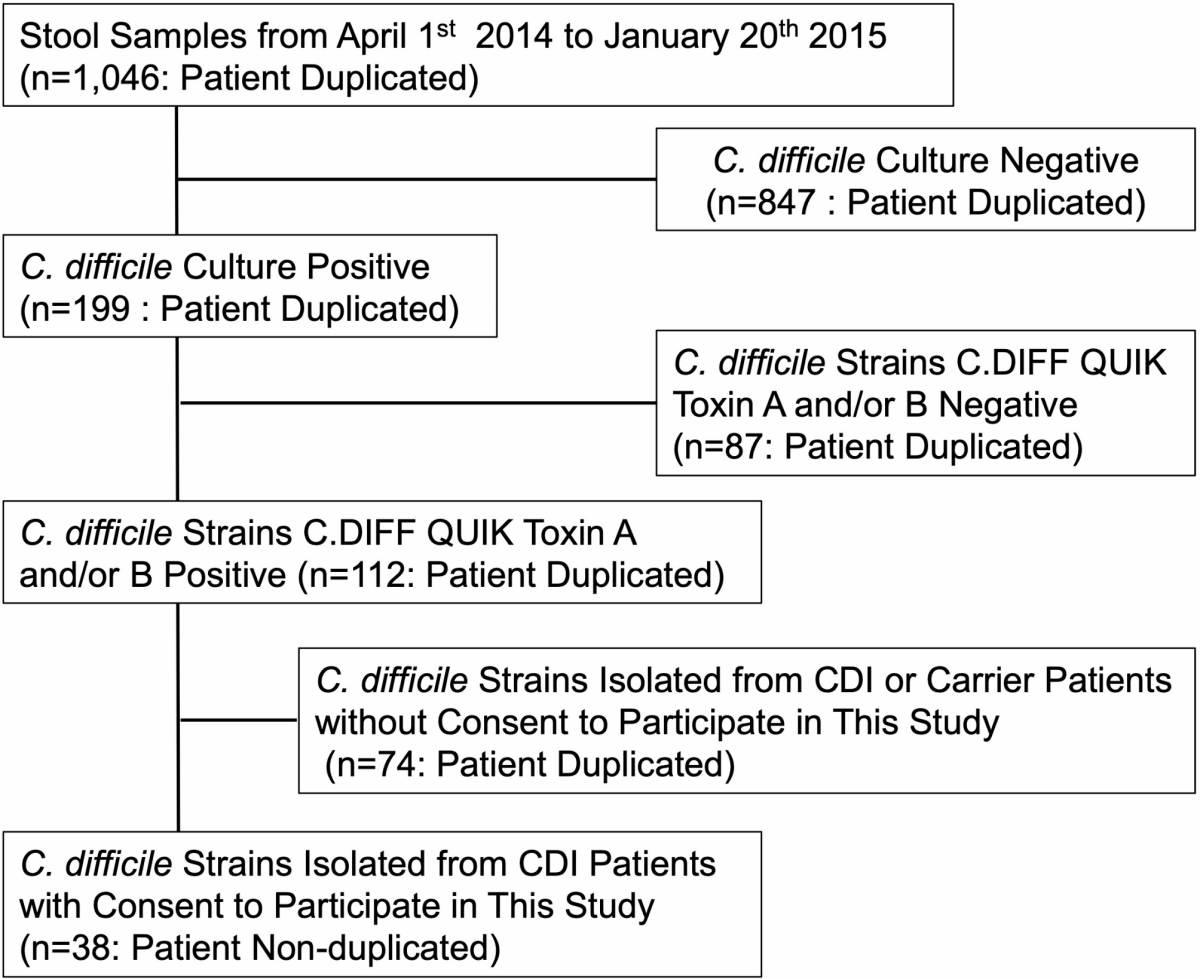
Sampling workflow of *C. difficile* strains in this study

This study was conducted as part of the Asian Pacific *C. difficile* Surveillance Study [6, 19]. Thirty-eight patients were included in the study in Fig. 1. Briefly, 1,046 stool samples collected for various medical purposes, including CDI diagnosis (note: there were instances of patient duplication), were submitted to the microbiological laboratory at Toho University Omori Medical Center from April 1st, 2014, to January 20th, 2015. *C. difficile* was isolated from 199 of these stools using ChromID™ *C. difficile* agar (bioMérieux, France) and incubating at 35 °C for 48 h. The presence of *C. difficile*, including both glutamate dehydrogenase and toxin A or B, was confirmed in 112 strains using an enzyme immune assay with C. DIFF QUIK COMPLETE^®^ (Kohjin Bio Co., Ltd., Saitama, Japan) and bacterial cell suspensions. Informed consent was obtained from 38 cases diagnosed with CDI, and first isolate strains from each case during the study period were used for this study (Table S1).

### Draft whole-genome sequencing analysis

DNA was extracted using a combination of achromopeptidase treatment and phenol/chloroform, followed by purification with the Wizard^®^ SV Gel and PCR Clean-Up Kit System. DNA libraries were prepared for sequencing on the MiSeq platform (Illumina Inc., CA, USA) using the Nextera XT DNA Library Preparation Kit (Illumina) and the Illumina DNA Prep (M) Tagmentation Library Preparation Kit (Illumina). These libraries were sequenced using the MiSeq Reagent Kit V3-600 cycles, allowing 300 bp paired-end reads (Illumina Inc.). Draft genome contigs were generated by *de novo* assembly using SPAdes version 3.15.5 [20]. Genome annotation and species identification were performed using Fast Average Nucleotide Identity with type strain genomes facilitated by the DNA Data Bank of Japan’s Fast Annotation and Submission Tool [21]. Gene identification and alignment analysis for the following genes: *tcdA* encoding toxin A (TcdA); *tcdB* encoding toxin B (TcdB); *cdtA* encoding binary toxin A (CdtA); *cdtB* encoding binary toxin B (CdtB); *tcdC* encoding the negative regulator of the *tcdA* and *tcdB*, were performed using Nucleotide BLAST [22] and Jalview version 2 [23]. *C. difficile* strain 630 (*tcdA* + *tcdB* + *cdtA*/*cdtB*-, accession no. NC_009089) and strain CD196 (*tcdA* + *tcdB* + *cdtA*/*cdtB*+, accession no. NC_013315) were used as reference genome of toxin gene sequences. MLST was performed using *C. difficile* MLST databases in PubMLST.org (https://pubmlst.org/cdifficile/). The draft WGS data has been deposited in the DDBJ/ENA/GenBank under BioProject accession number PRJNA1036794, with individual sample accession numbers listed in Table S1.

### Core-genome SNPs-based phylogenetic analysis

Phylogenetic analysis based on core-genome SNPs was performed according to our previous report [11]. Briefly, sequencing reads were aligned to the reference genomes of genetically closest strains for each ST using the Burrows-Wheeler Aligner with the ‘SW’ algorithm [24]. The core-genome sequence were extracted using the Sequence Alignment/Map (SAMtools) software, version 1.1 [25], with the “mpileup” option, and VarScan version 2.3.7, using the “mpileup2cns” option [26]. The homologous recombination regions were estimated using ClonalFrameML and were excluded from the core-genome. A phylogenetic tree was generated using RAxML, based on SNPs within the core-genome, excluding the homologous recombination regions.

## Results

### Draft whole-genome sequencing and molecular characterizing of *C. difficile*

Results and characteristics of the draft WGS are presented in Table S1. MLST classified 38 strains into 11 STs. The most prevalent were strains belonging to ST81 (*n* = 11), followed by ST183 (*n* = 10) and ST17 (*n* = 7), as shown in Table 1. Strains from other STs were detected in fewer than two instances. ST81 strains were isolated from patients across nine wards, whereas ST183 and ST17 were predominantly found in 3 wards (Table 1). Specifically, of the 10 ST183 strains, eight were isolated from patients in Ward B, which is the Department of Hematology and Oncology. Similarly, of 7 ST17 strains, five were from Ward D, the Department of Respiratory Medicine. Toxin type varied depending on the ST, except for ST5. Strains of ST183 and ST17 were characterized as A + B + CDT-, while ST81 was A-B + CDT-, as detailed in Table S1.

**Table 1:** Sequence type of *C. Difficile* and the hospital wards where the patients were admitted

	Hospital ward											
MLST	A	B	C	D	E	F	G	H	I	J	K	Total
ST81	1	2	1	1	2		1		1	1	1	**11**
ST183	1	8									1	**10**
ST17				5						1	1	**7**
ST5			1				1					**2**
ST8						1		1				**2**
ST2				1								**1**
ST3								1				**1**
ST37			1									**1**
ST42										1		**1**
ST100								1				**1**
ST185	1											**1**

### Core-genome SNPs-based phylogenetic analysis

**Fig. 2:**
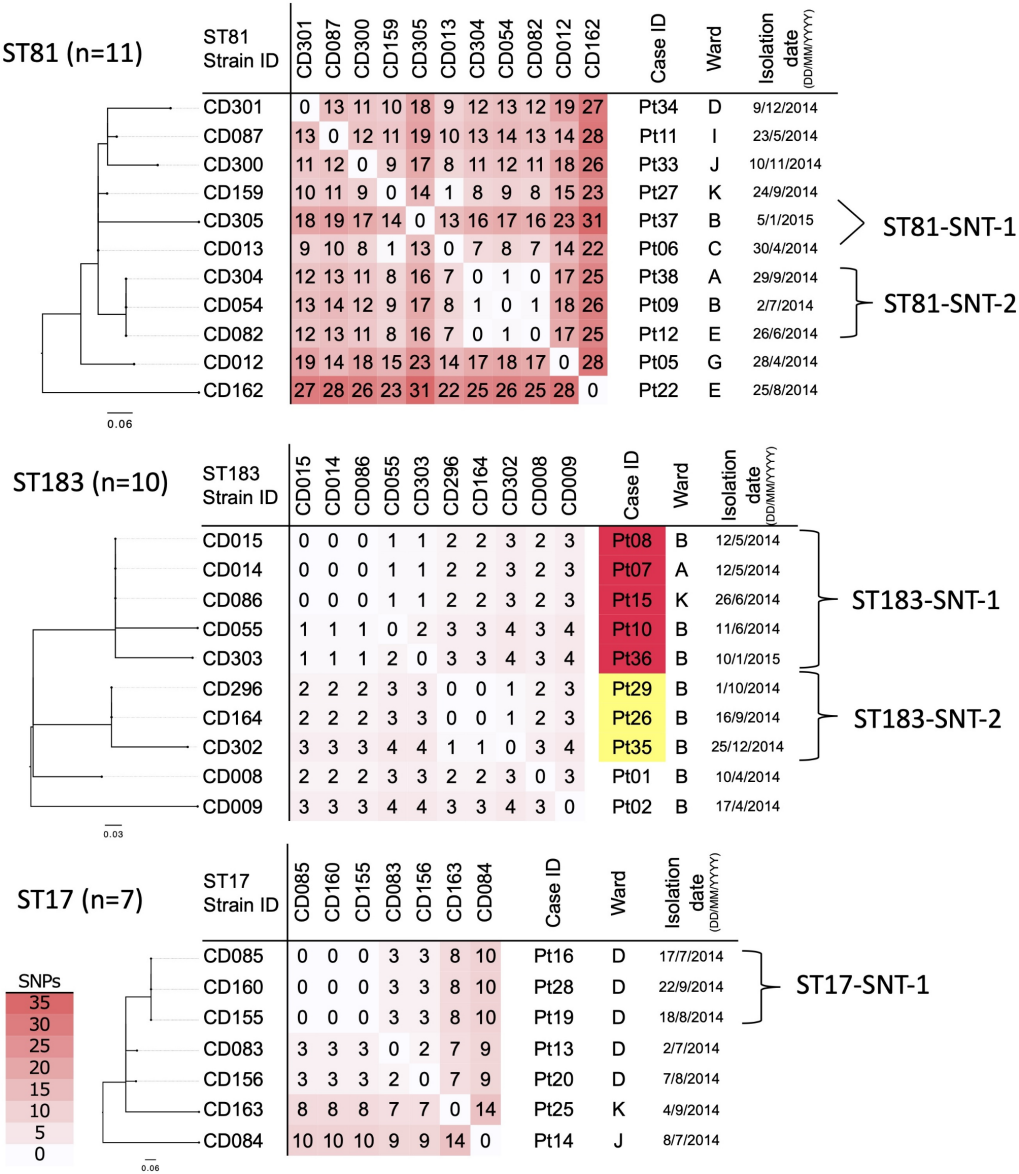
Phylogenetic analysis of major sequence types (STs) of *C. difficile* based on core-genome single nucleotide polymorphisms (SNPs). The number of SNPs is displayed in a matrix. The core-genome size rate (%), defined as the ratio of the core-genome size to the reference genome, is presented as follows; 65.9% for ST81, calculated as 2,830,796/4,293,712 bp (reference CE91-St50 [ST81, NZ_AP025558.1]); 49.1% for ST183, calculated as 2,008,677/4,089,134 bp (reference S-0253 [ST8, NZ_CP076401.1]); 29.9% for ST17, calculated as 1,230,694/4,109,635 bp (reference FDAARGOS_267 [ST3, NZ_CP020424.2]). Notably, strains of Suspected Nosocomial Transmission (SNT) are highlighted in red (for ST183-SNT-1) and yellow (for ST183-SNT-2), corresponding to Fig. 3

The rate of core-genome size relative to reference genome size was 65.9% for ST81, 49.1% for ST183, and 29.9% for ST17. We established a distinct cutoff for Suspected Nosocomial Transmission (SNT) for each ST due to their varying core-genome size: ≤3 SNPs for ST81, ≤ 2 SNPs for ST183, and SNPs ≤ 1 for ST17. Based on these criteria, two SNT groups were identified in ST81 (ST81-SNT-1 comprising CD013 and CD159; ST81-SNT-2 comprising CD054, CD082, and CD304), two in ST183 (ST183-SNT-1 comprising CD014, CD015, CD055, CD086, and CD303; ST183-SNT-2 comprising CD164, CD296, and CD302), one in ST17 (ST17-SNT-1 comprising CD085, CD155, and CD160), as shown in Fig. 2. In ST183, the number of SNPs detected across all strains was fewer than in ST81 and ST17 (< 5 SNPs). The retrospective draft WGS identified five clusters, and 16 of 38 strains (42.1%) were genetically related to any of them.

### Moving patients between wards in the hospital

**Fig. 3:**
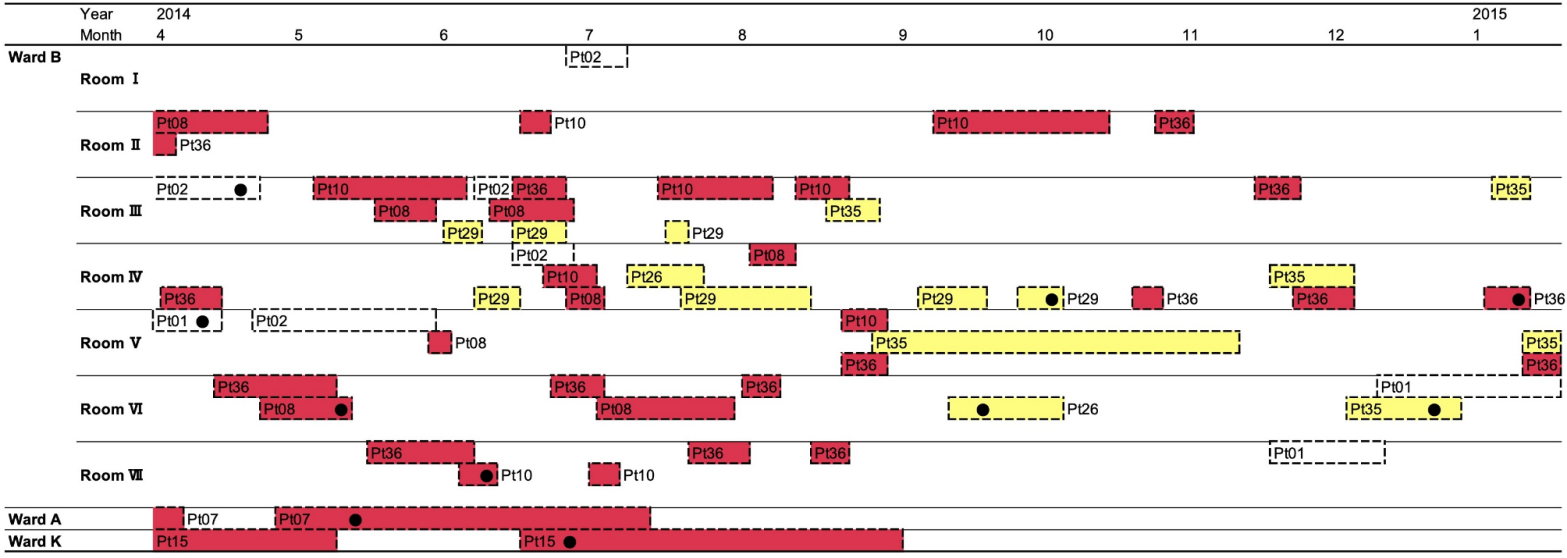
Information on hospital rooms of patients from whom *C. difficile* ST183 was isolated. The dashed squares indicate the hospitalization periods for each patient. Filled circles mark the dates when *C. difficile* was isolated from these patients. Patients who have suspected nosocomial transmission (SNT) of *C. difficile* ST183 are highlighted in red for ST183-SNT-1 and in yellow for ST183-SNT-2, as referenced in Fig. 2

ST17 was isolated consistently over a three-month period, whereas ST183 was isolated sporadically over a ten-month period. Our focus was on ST183, for which we visualized the hospitalization periods and room assignments of patients in Ward B (Fig. 3). Patients associated with ST183-SNT-1, which comprised five individuals, had stayed in 6 rooms within Ward B, one room in Ward A, and another in Ward K. Those associated with ST183-SNT-2, consisting of 3 patients, had stayed in 4 different rooms within Ward B. Notably, patients linked to both ST183-SNT-1 and ST183-SNT-2 frequently moved rooms within the ward. Pt08, who belonged to ST183-SNT-1 and moved room 7 times, was the most frequent mover. Pt36, also a part of ST183-SNT-1, was hospitalized and discharged five times. Pt10 moved rooms five times, was then discharged, re-hospitalized, and subsequently moved rooms an additional two times. Pt08, Pt10, and Pt36 had multiple overlaps in room occupancy during different periods. Patients of ST183-SNT-2 were frequently changing rooms, though they did not often share rooms.

## Discussions

We identified silent and multiple nosocomial transmission of *C. difficile* through draft WGS of isolates from additional culture tests over a period of 10 months in a single center. These patients frequently moved between rooms within the ward and experienced repeated discharges and re-hospitalizations. Implementing aggressive additional culture tests for *C. difficile* and conducting retrospective WGS analysis of the isolated strains could be instrumental in detecting and monitoring the effectiveness of routine infection control measures.

In this study, the main genetic lineage identified were ST81, ST183, and ST17, which have been reported as the top five frequently isolated lineages in Japan [11]. Patients isolated with ST183 were suggested to have higher risks for acquiring or transmitting *C. difficile*, potentially due to frequent room changes. This is supported by a previous report indicating that patients staying in the same room as a CDI patient have a higher risk of developing CDI [27]. Patients with CDI received antimicrobial treatment, and half of them continued to carry *C. difficile.* Moreover, their skin and surroundings could be contaminated with *C. difficile* [28].

The SNP accumulation rate of *C. difficile* is reported to be approximately 1.7 × 10^− 6^ mutations per site per year [29]. This translates to about one SNP emerging every two months, or seven SNPs per year, based on the genome size of *C. difficile* type strain ATCC 9689 = DSM 1296 (GenBank accession number CP011968.1), which is 4,109,692 bp. The core-genome rate, or the core-genome size relative to the reference genome size, is crucial when interpreting genetic relationships among bacterial strains. In our previous report, the core-genome rate was 58.4% for ST17, 32.0% for ST8, 34.1% for ST2, 72.6% for ST81, and 41.8% for ST183, using the *C. difficile* strain CD630 belonging to ST54 (accession number: NC_009089) as a reference genome. This study found no major differences in ST81 (65.9%) and ST183 (49.1%) but approximately half in ST17 (29.9%). The absence of complete genome sequence data for *C. difficile* ST17 in GenBank, and the closest hit being a ST3 strain genome in MINTyper analysis, suggests a genetic distance between ST17 and ST3 as a possible reason for the small core-genome rate in ST17. Therefore, the cut-off for interpreting core-genome SNP analysis results should be variable and dependent on the core-genome rate. In other words, it is important to note that if the core-genome size is halved, the detectable SNPs may also be halved. We adjusted the criterion for the number of SNPs suspected of transmission according to the core-genome rate by ST to avoid overestimating low SNP numbers.

This retrospective study offers an opportunity to consider the silent transmission of *C. difficile* in a hospital. A previous study conducted in a hospital in China showed a similar genetic relation rate among included *C. difficile* strains, with 43.8% (110/241 strains) being comparable to the 42.1% (16/38 strains) found in this study [30]. Although *C. difficile* ST17 was isolated within a short period, it was not considered a case of nosocomial transmission in the ward. Similarly, in the case of ST183, predicting nosocomial transmission would be more challenging when considering the accumulation of *C. difficile* isolates from patients across multiple wards over an extended period. Most patients were hospitalized in rooms shared with multiple other patients, which likely led to a higher frequency of care from common medical staff, thereby increasing the risk of transmitting *C. difficile*. The frequent events of patients moving rooms could have contributed to an increase in the provision of medical care.

This study has three limitations. First, it is a single-center, retrospective observation study. Our focus was on observing nosocomial transmission of *C. difficile*, and we did not collect information on CDI or the administration of antimicrobial agents. Additionally, we did not perform screening cultures for *C. difficile* on asymptomatic patients staying in the same room as CDI patients. Second, the impact of analyzing silent transmission on infection control and its contribution to healthcare economics was not assessed in this study. Third, we analyzed only one strain, specifically the first one isolated from each patient during the study period. It is important to consider, when interpreting the results that a patient may carry polyclonal *C. difficile*, encompassing several genetically unrelated strains belonging to different STs [31–33].

## Conclusion

Additional culture tests for *C. difficile* and draft WGS has enabled the detection of *C. difficile* transmission that was previously overlooked. Analyzing *C. difficile* transmission could serve as a valuable marker for monitoring the effectiveness of infection control practices in hospitals.

## Electronic supplementary material

Below is the link to the electronic supplementary material.

Supplementary Material 1

## Data Availability

Draft WGS data have been deposited in the GenBank BioProject under the accession number PRJNA1036794.

## Abbreviations

CDI: Clostridioides difficile infection
RT: Ribotyping
MLST: Multilocus sequencing typing
SNP: Single nucleotide polymorphism
EIA: Enzyme immune assays
NAAT: Nucleic acid amplification testing
TcdA: Toxin A
TcdB: Toxin B
CdtA: Binary toxin A
CdtB: Binary toxin B
